# Supplementary material for: Superoxide Dismutase-3 Downregulates Laminin α5 Expression in Tumor Endothelial Cells via the Inhibition of Nuclear Factor Kappa B Signaling
Source: Cancers (Basel). 2022 Feb 26;14(5):1226. doi: 10.3390/cancers14051226 (PMC8909228; doi:10.3390/cancers14051226)
Supplement: Supplementary file 1 [file cancers-14-01226-s001.zip › cancers-1555593-supplementary.pdf]

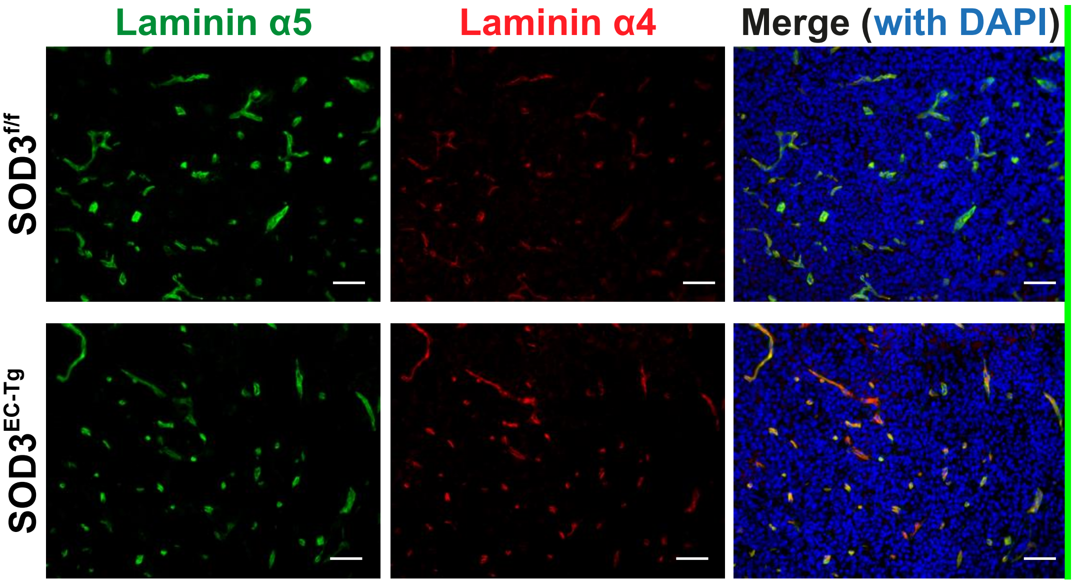

Figure S1. Differential regulation of laminin α5 and laminin α4 by SOD3 in tumors. Low magnification images of EG7 tumor sections grafted in SOD3$^{EC\text{-}Tg}$ and SOD3$^{f/f}$ mice, stained with laminin α5 (green) and laminin α4 (red) as described in Figure 1G; nuclei were DAPI counterstained (blue). Scale bars 50 μm.

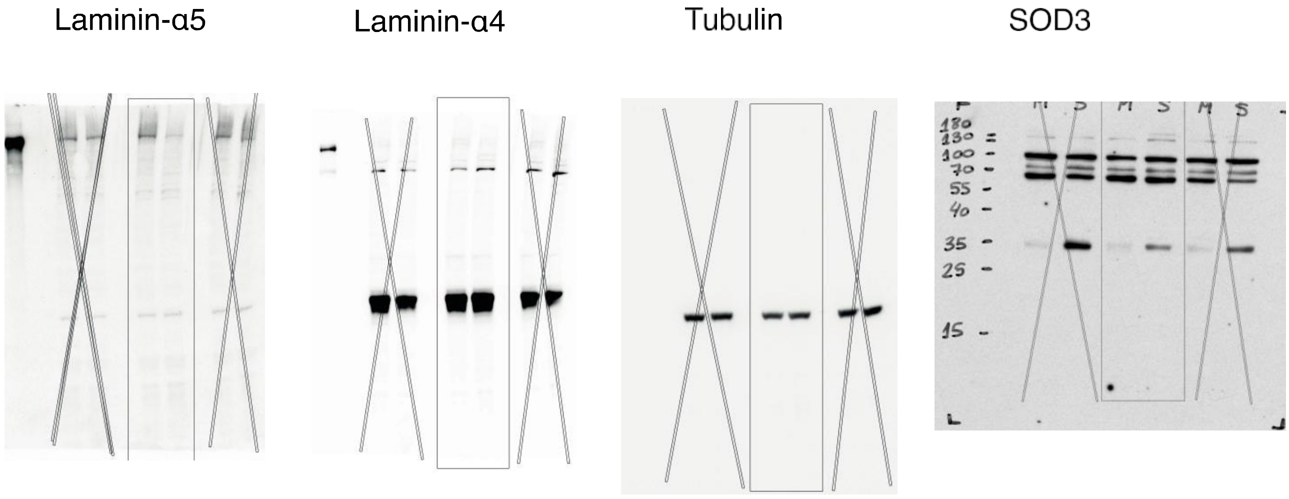

Figure S2. Original immunoblots for Figure 2D

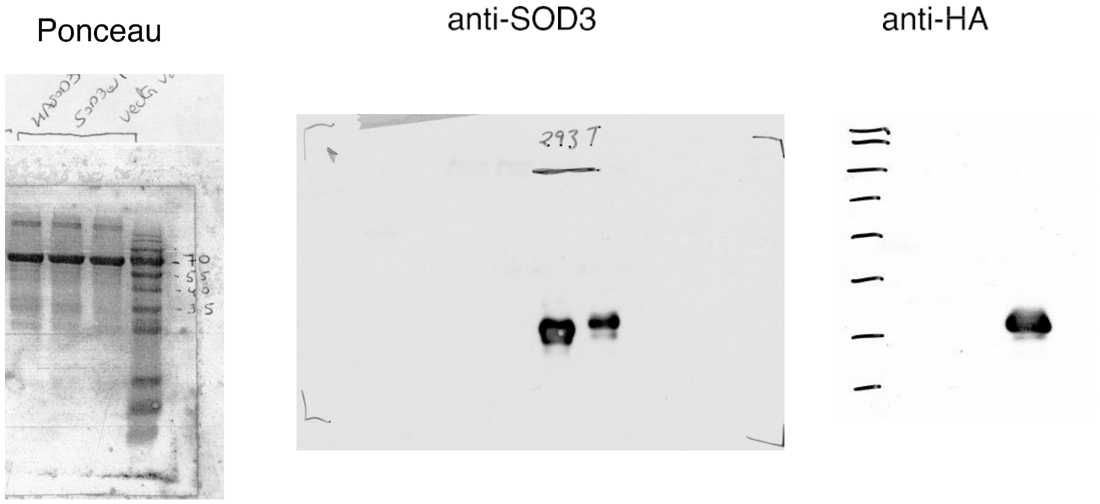

Figure S3. Original immunoblots for Figure 2G.

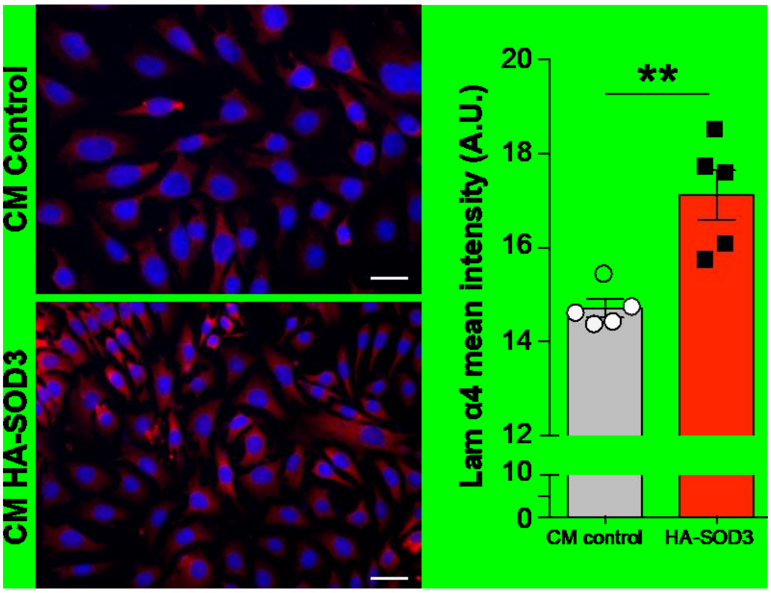

Figure S4. Extracellular SOD3 upregulates laminin $\alpha 4$. (**A**) 1G11 cells were incubated with control or HA-SOD3 conditioned medium and stained with anti-laminin $\alpha 4$ antibodies (red); nuclei were DAPI-counterstained (blue). Scale bars 50 µm. (**B**) Quantification of laminin $\alpha 4$ mean fluorescence intensity from *A* (n=5 fields/condition). **P<0.01; two-tailed *t*-test.

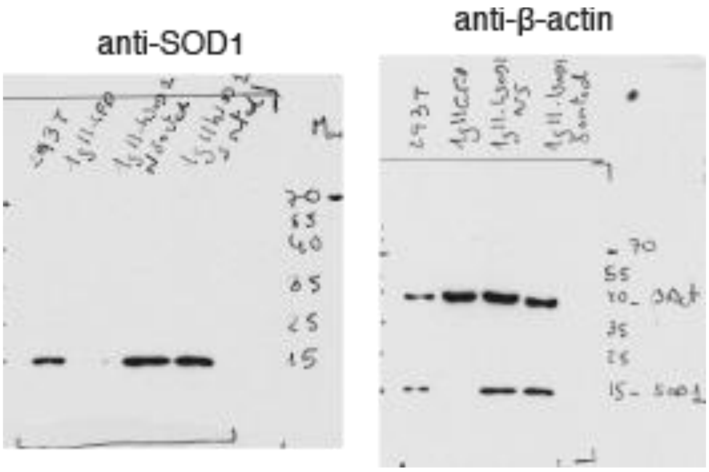

Figure S5. Original immunoblots for Figure 3B.

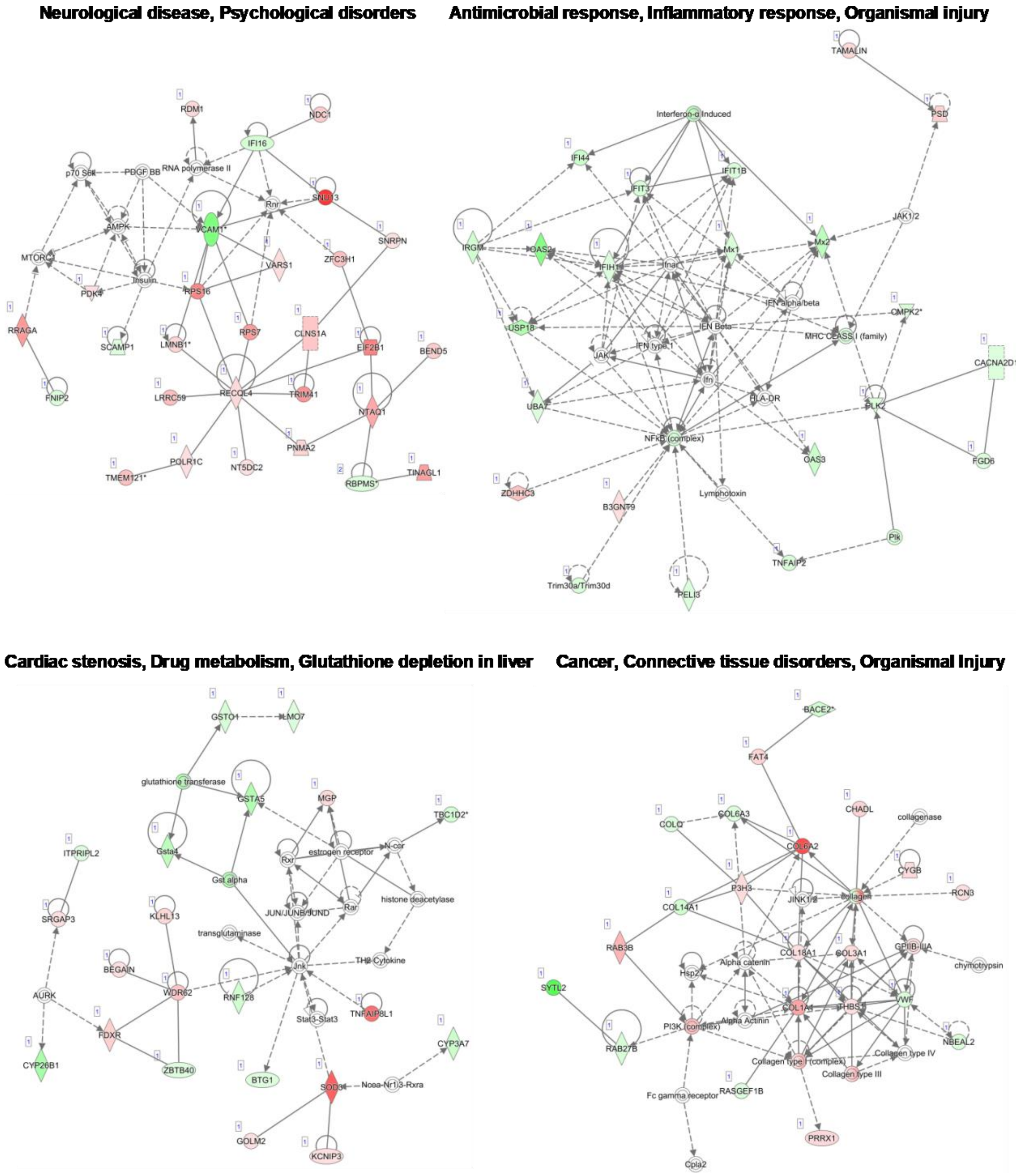

Figure S6. Molecular networks with an IPA score of >30. Colors represent the downregulation (green) or upregulation (red) in 1G11-SOD3 *vs.* 1G11-mock cells.

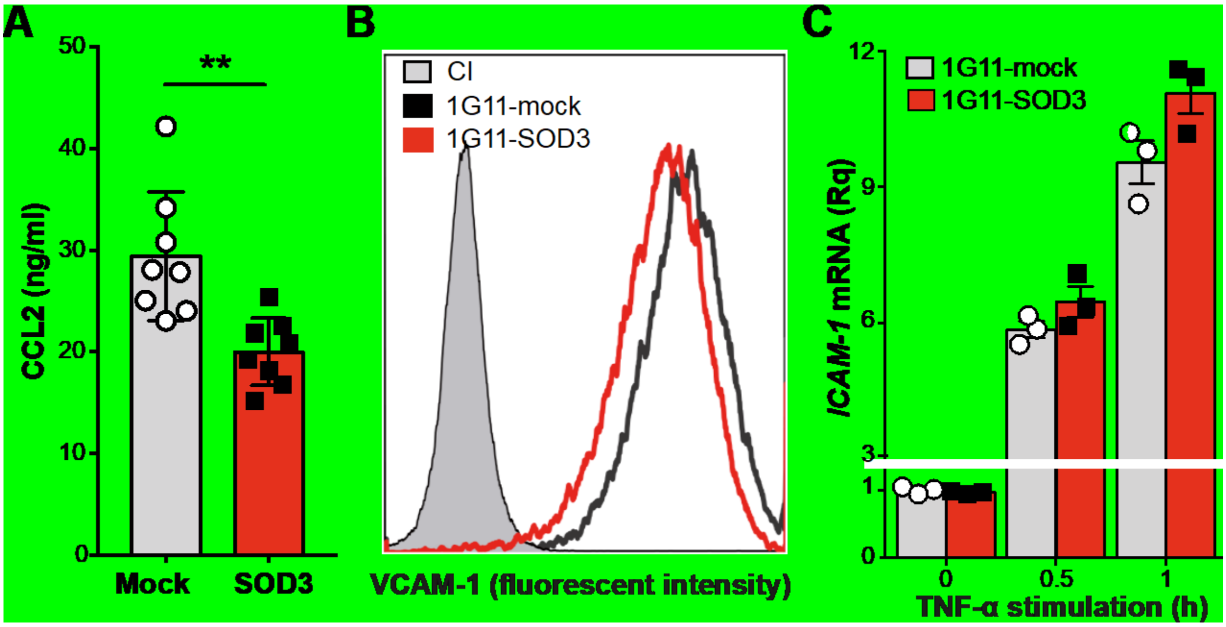

Figure S7. Effect of SOD3 overexpression in CCL2, VCAM-1 and ICAM-1 levels. (**A**) Quantification of CCL2 in the supernatant of unstimulated 1G11-mock and 1G11-SOD3 cells by ELISA ($n = 8$). **$P < 0.01$, two-tailed *t*-test. (**B**) Determination of VCAM-1 levels at the cell surface of unstimulated 1G11-mock (black) and 1G11-SOD3 cells (red) by flow cytometry; the staining with an isotype control antibody is shown in gray (negative control). A representative histogram is shown ($n = 3$). (**C**) 1G11-mock and -SOD3 were stimulated with TNF-α for the indicated times and the relative expression of I*CAM-1* determined by RT-qPCR ($n = 3$ independent experiments). Unstimulated condition (time 0). No significant differences were found between mock- and SOD3-expressing cells (Student's *t*-test).

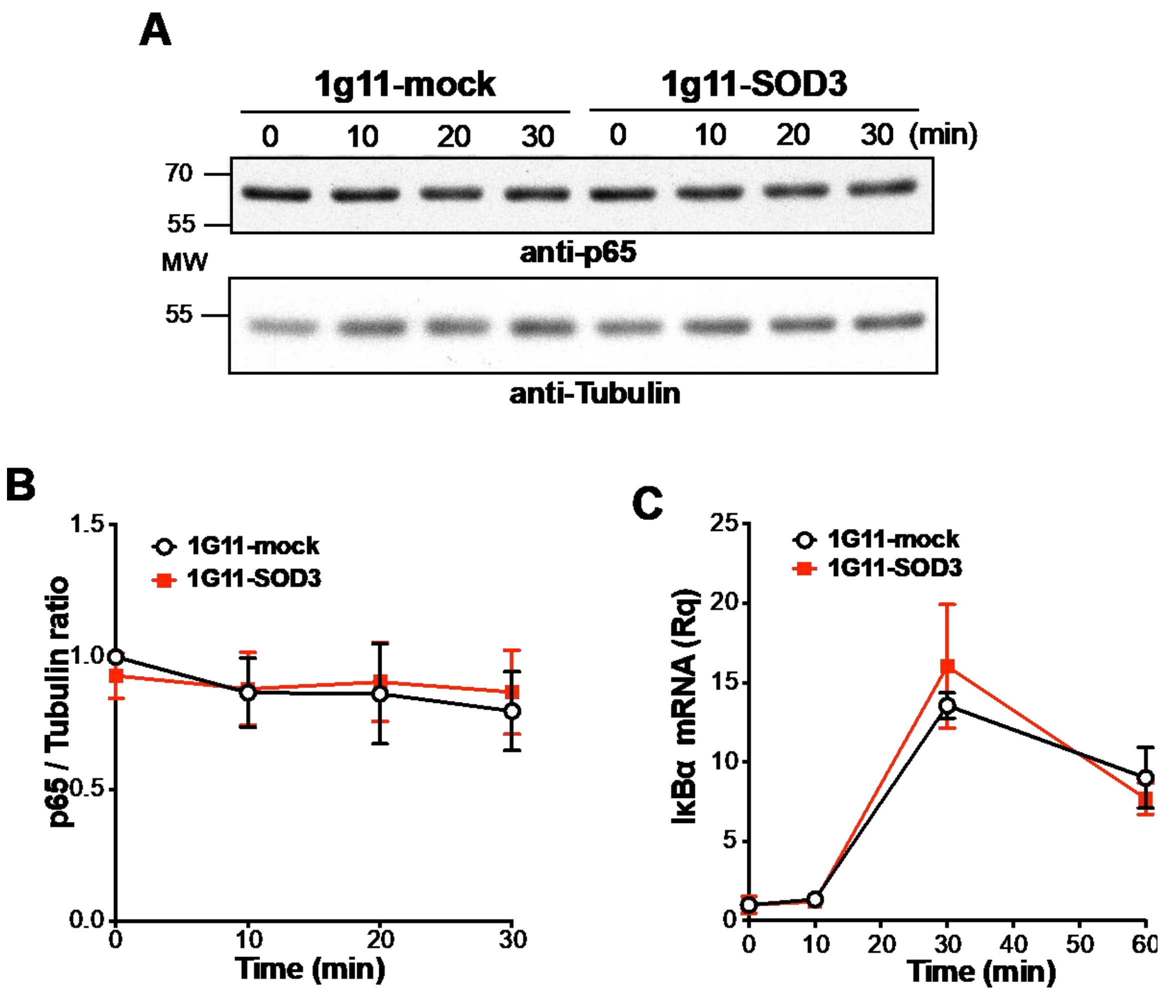

Figure S8. p65 protein levels and relative IκBα mRNA expression in 1G11-mock and 1G11- SOD3 cells. (**A**) Representative immunoblot of cell extracts from 1G11-mock and 1G11-SOD3 cells at different times after TNF-α stimulation. Filter were sequentially hybridized with anti- p65 (upper) and anti-α-tubulin (bottom; loading control) antibodies. (**B**) Densitometric quantification of blots as in (**A**). Data are mean±SEM (n = 3); no significant differences between 1G11-mock and 1G11-SOD3 cells were detected (two-tailed Student's *t*-test). (**C**) Relative quantity of IκBα mRNA levels in TNF-α-stimulated 1G11-mock and 1G11-SOD3 cells. Values were normalized with the corresponding unstimulated condition (time 0). As reported, TNF-α induced IκBα expression but no significant differences were observed between 1G11-mock and.-SOD3 cells (two-tailed Student's *t*-test). Data are mean ± SEM (n = 6).

anti-IκB-α

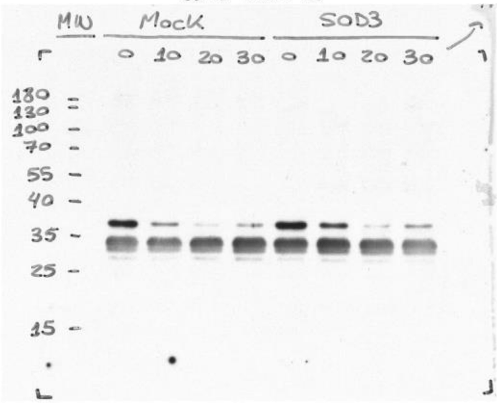

Figure S9. Original immunoblots for Figure 6A.

anti-Tubulin

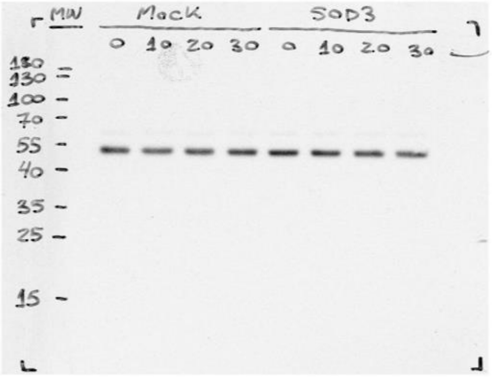

Figure S10. Original immunoblots for cropped images in Figure 6C.

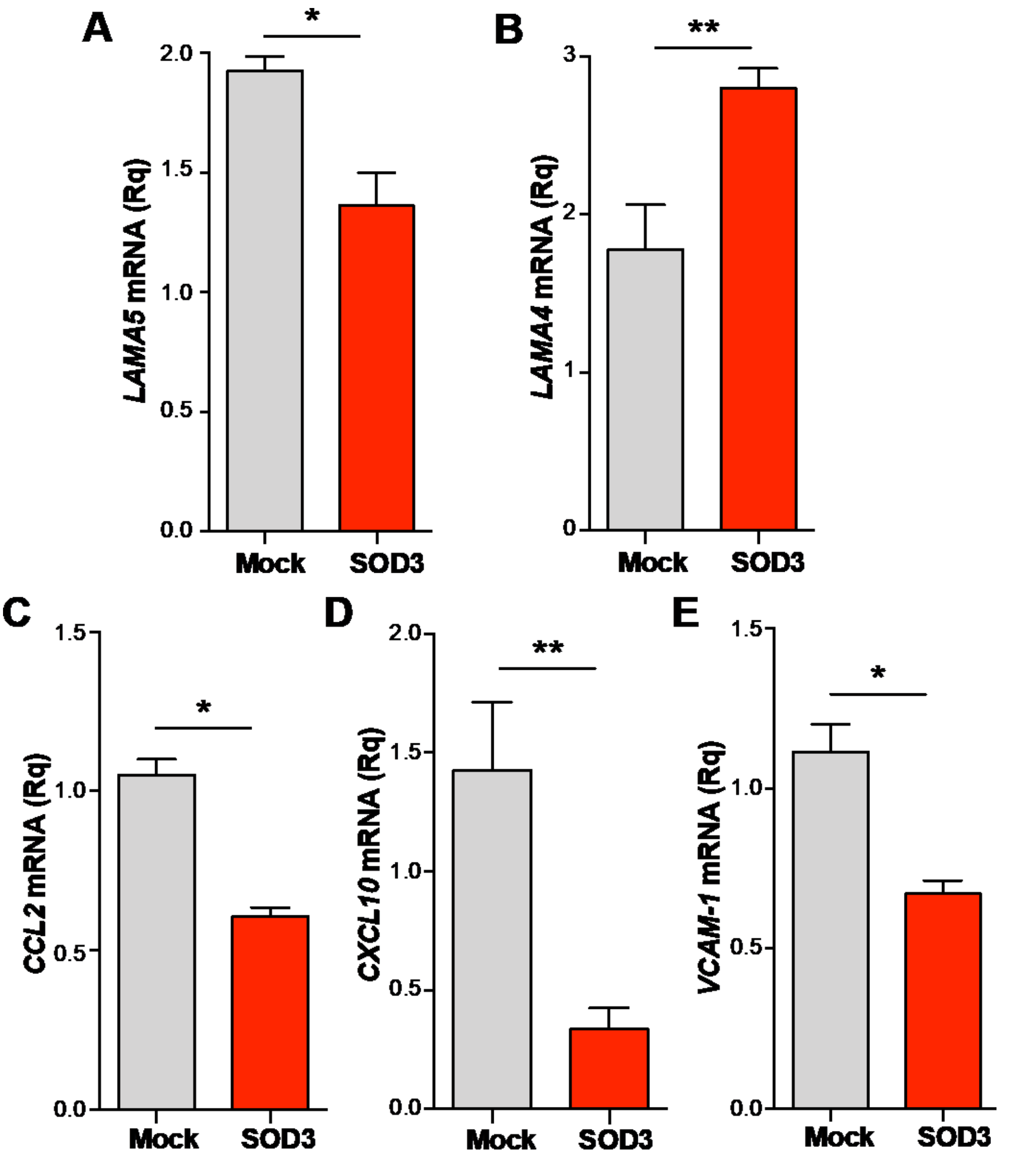

Figure S11. Relative expression of laminins and NF-κB target genes in SOD3-overexpressing human endothelial cells. Human dermal microvascular endothelial cells (HDMEC) were transduced with adenoviruses encoding for β-galactosidase (mock) or human SOD3. It is shown the relative expression of (**A**) *LAMA5*, (**B**) *LAMA4*, (**C**) *CCL2*, (**D**) *CXCL10* and (**E**) *VCAM-1* by RT-qPCR. Data are mean±SEM of triplicates in n=3 independent experiments. * $P < 0.05$, ** $P < 0.01$, two-tailed Student's t-test.

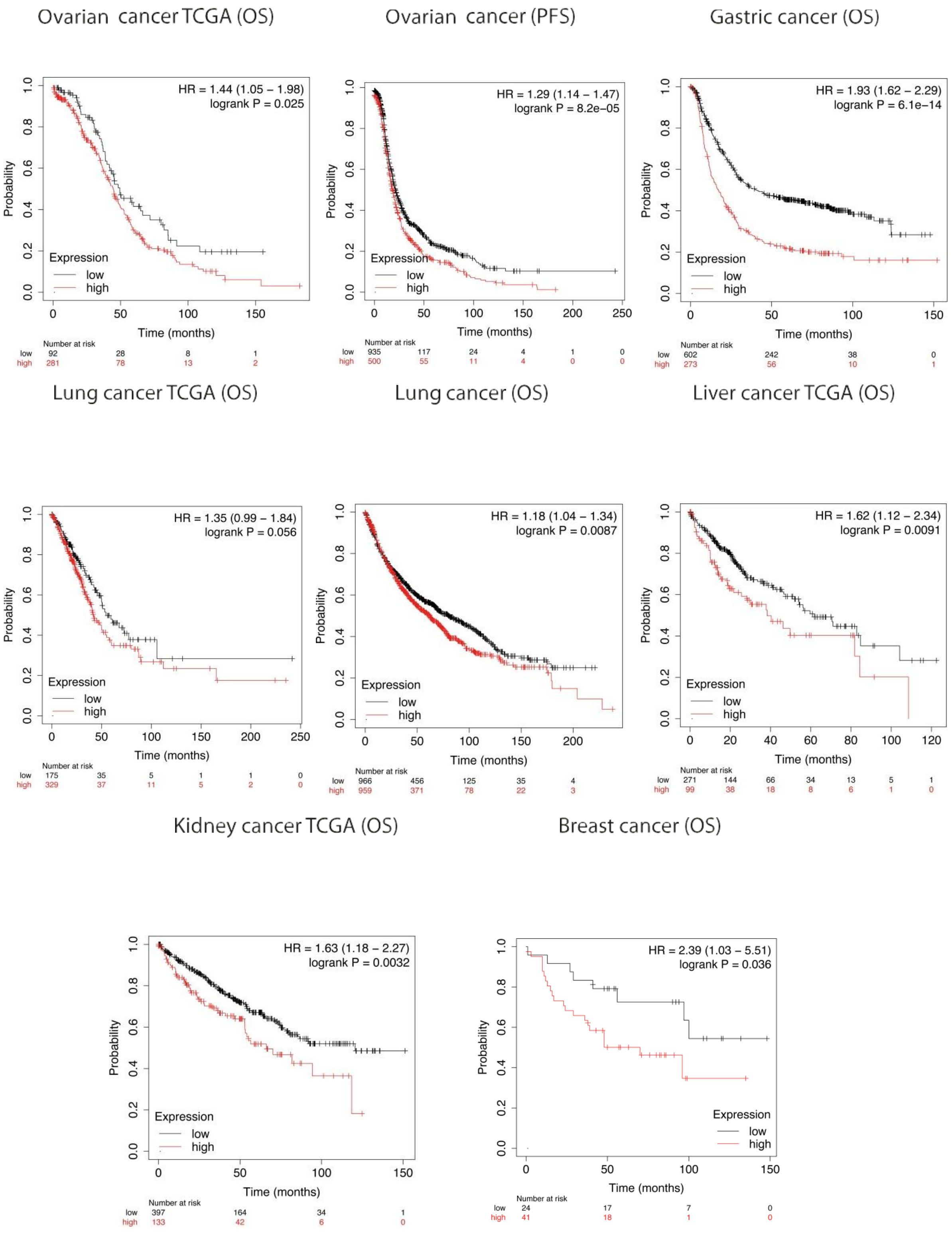

Figure S12. Association of laminin $\alpha5$ levels with the prognosis of different cancers. Kaplan- Meier curves for cumulative overall survival (OS) or progression-free survival (PFS) for patients with the indicated cancers based on high and low laminin $\alpha5$ levels. The curves were obtained using the Kaplan-Meier plotter database. The cutoff was selected automatically by the algorithm to provide the best cutoff in each platform. The p-value of the difference for each comparison was obtained using the log-rank test. The hazard ratio (HR) is also shown for each comparison. Breast cancer data were based on laminin $\alpha5$ protein levels, whereas the other comparisons were based on mRNA levels from TCGA or GEO databases. The comparison from different databases is shown for ovarian and lung cancer datasets, suggesting a reasonable parallelism in the prediction.

Table S1. Primers used for RT-qPCR for mouse (m) and human (h) genes.

Gene	Forward primer (5'-3')	Reverse primer (5'-3')
**mLAMA5**	GGCCAGGAAGAACCAGCTA	GCAATCTTCTCACTGGTCTCG
**hLAMA5**	CCTCGTCCTCCAATGACAC	GCGCTGCAGTCACAATTC
**mLAMA4**	CACGTGACCGACATGAACTC	TTCTCTTTCTGACAGCCTTGTTC
**hLAMA4**	GGATGCCGAAGACATGAAC	TTCCCTCACTCTTTCCTGTTGT
**mCCL2**	TCTGGGCCTGCTGTTCACA	TTGGGATCATCTTGCTGGTG
**hCCL2**	AGACTAACCCAGAAACATCC	ATTGATTGCATCTGGCTG
**mCXCL10**	TGCTGGGTCTGAGTGGGACT	CCCTATGGCCCTCATTCTCAC
**hCXCL10**	GTGGCATTCAAGGAGTACCTC	GCCTTCGATTCTGGATTCAGACA
**mFAS**	AAACCAGACTTCTACTGCGATTCT	GGGTTCCATGTTCACACGA
**mICAM-1**	GTGGCGGGAAAGTTCCTG	CGTCTTGCAGGTCATCTTAGGAG
**mVCAM-1**	TCTTACCTGTGCGCTGTGAC	ACTGGATCTTCAGGGAATGAGT
**hVCAM-1**	TGCACAGTGACTTGTGGACATA	GCCACCACTCATCTCGATTT
**mIκB-α**	AAGGACGAGGAGTACGAGCAA	GGATGATTGCCAAGTGCAGGA
**mACTB**	GAGAAACGGCTACCACATCC	GGGTCGGGAGTGGGTAAT
**hACTB**	CCCAGCACAATGAAGATCAA	CGATCCACACGGAGTACTTG
**hSOD1**	TCATCAATTTCGAGCAGAAGG	CAGGCCTTCAGTCAGTCCTTT

Table S2. Correlation between *LAMA5* and CD8 T cell infiltration in human cancers.

Cancer type	Spearman's correlation	*p*-value
Head-Neck squamous cell carcinoma	–0.11	2.94e–02
Lung adenocarcinoma	–0.14	1.96e–03
Lung squamous carcinoma	–0.259	9.87e–09
Ovarian cancer	–0.186	4.29e–05
Skin cutaneous melanoma	–0.083	8.27e–02
Stomach adenocarcinoma	–0.199	1.13e–04
Thyroid cancer	–0.449	1.71e–25
Thymoma	–0.354	1.13e–04
Uterine corpus endometrial carcinoma	–0.21	3.26e–04
